# Phytochemicals and Antioxidant Activity of Korean Black Soybean (*Glycine max* L.) Landraces

**DOI:** 10.3390/antiox9030213

**Published:** 2020-03-05

**Authors:** Kyung Jun Lee, Da-Young Baek, Gi-An Lee, Gyu-Taek Cho, Yoon-Sup So, Jung-Ro Lee, Kyung-Ho Ma, Jong-Wook Chung, Do Yoon Hyun

**Affiliations:** 1 National Agrobiodiversity Center, National Institute of Agricultural Sciences (NAS), RDA, Jeonju 54874, Korea; lkj5214@korea.kr (K.J.L.); gtcho@korea.kr (G.-T.C.); jrmail@korea.kr (J.-R.L.); 2 Department of Crop Science, Chungbuk National University, Chungdae-ro 1, Seowon-Gu, Cheongju, Chungbuk 28644, Korea; dayung96@naver.com (D.-Y.B.); yoonsupso@chungbuk.ac.kr (Y.-S.S.); 3 Department of Herbal Crop Research, NIHHS, RDA, Eumseong 27709, Korea; khma@korea.kr; 4 Department of Industrial Plant Science and Technology, Chungbuk National University, Chungdae-ro 1, Seowon-Gu, Cheongju, Chungbuk 28644, Korea

**Keywords:** antioxidant activity, black soybean, *Glycine max*, Korean landraces, phytochemicals

## Abstract

Black soybean (*Glycine max* L.) has been used as a traditional medicine because its seed coat contains various natural phenolic compounds such as anthocyanins. The objective of this study was to reveal the genetic variation in the agricultural traits, phytochemicals, and antioxidant activity of 172 Korean black soybean landraces (KBSLs) and establish a relationship among them. The evaluation of three agricultural traits (days to 50% flowering, maturity, and 100-seed weight), six phytochemicals (delphinidin-3-glucoside, cyaniding-3-glucoside, petunidin-3-glucoside, daidzin, glycitin, and genestin), and four antioxidant activities (2,2-diphenyl-1-picrylhydrazyl (DPPH), 2,2′-azino-bis-(3-ethylbenzothiazoline-6-sulfonic acid)(ABTS), ferric-reducing antioxidant power (FRAP), and the total polyphenol content (TPC) of 172 KBSLs were analyzed in 2012 and 2015. The agricultural traits, phytochemicals, and antioxidant activities of the 172 KBSLs showed wide variation among the accessions and years. In correlation analysis, the agricultural traits and phytochemicals showed positive and negative correlations with phytochemicals and antioxidant activity, respectively. The principal component analyses result indicated that phytochemicals accounted for most of the variability in the KBSLs. In clustering analysis, the 172 KBSLs were classified into four clusters. These results could lead to expanding the knowledge of the agricultural traits, phytochemicals, and antioxidant activity of the KBSLs, which are valuable materials for the development of new soybean varieties.

## 1. Introduction

Soybean (*Glycine max* L. Merr.) is widely cultivated and consumed throughout the world as grains, tofu, and soy milk [1]. It contains 40% protein and 20% lipid, which mainly consists of unsaturated fatty acids such as oleic acid and linoleic acid [2]. In addition, soybean has various compounds beneficial to health, such as isoflavone, oligosaccharide, saponins, and phenolics [3,4]. These reportedly have preventive effects on cancer, cardiovascular disease, obesity, and diabetes [1].

Soybean coat color is an important attribute determining the outward appearance of the soybean seed, which exists in a range of colors from yellow, green, brown, and black, to bicolored [5]. Among them, black soybean has been used in folk medicine in China, India, Japan, and Korea for hundreds of years [6]. Recently, black soybeans have been found to contain high contents of γ-tocopherol, isoflavones, flavonoids, and anthocyanins with biological activity [7]. Antioxidant properties resulting from the free-radical-scavenging activity and total phenolic compounds are higher in black soybeans compared to their yellow counterparts [8].

Phytochemicals are natural bioactive components that are abundant in foods such as whole-grain products, legumes, tea, and dark chocolate [9]. The most common phytochemicals in food include polyphenols, flavonoids, isoflavones, phenolic acids, stilbenoids, isothiocyanates, saponins, procyanidins, phenylpropanoids, anthraquinones, ginsenosides, and others [10,11]. In plants, the accumulation of phytochemicals is reportedly influenced by environmental factors and growth conditions. Alterations in the accumulation of phytochemicals in plants may have adverse effects on the health benefits resulting from their consumption [12].

There is increasing interest in natural antioxidant products as medicines and food additives [13,14]. More recently, antioxidant compounds have received attention from natural-product consumers and researchers due to their pharmacological properties. Antioxidants lower the oxidative stress caused by reactive oxygen species (ROS) [15]. Phytochemicals, such as polyphenols and carotenoids, are important because of their contributions to human health and multiple biological effects including antioxidant, antimutagenic, anticarcinogenic, and cytoprotective activities [16].

Korean soybean landraces have large genetic variations because Korea has a significantly long history of domestication and cultivation of soybean [17,18]. They have been used as genetic resources in the US, Canada, China, and Japan to develop elite cultivars with high yield, disease resistance, and tolerance to environmental stresses [19]. Landrace, defined by the Camacho-Villa et al., is “as dynamic populations of a cultivated plant with a historical origin and distinct identity, often genetically diverse and locally adapted, and associated with a set of farmers’ practices of seed selection and field management, as well as with the farmers’ knowledge base” [20]. Dwivedi et al. [21] referred to plant landraces as heterogeneous local adaptations of domesticated species providing genetic resources for farming in stressful environments. In addition, landraces show variable phenology, guarantee stable edible yield, and are often nutritionally superior.

In this study, the agricultural traits, phytochemicals, and antioxidant activities in a set of 172 Korean black soybean landraces (KBSLs) were evaluated. The aim of this study was to reveal the genetic variation in 172 KBSLs to provide information on KBSLs which are valuable as a functional food crop and/or a new dietary ingredient.

## 2. Materials and Methods 

### 2.1. Plant Materials and Agricultural Traits

A total of 172 KBSLs were obtained from the National Agrobiodiversity Center of the Rural Development Administration (RDA), Republic of Korea. Each accession was sown with 20 seeds on May 25, 2012, at Suwon (37.275015, 126.984987) and on May 27, 2015, at Jeonju (35.831037, 127.062490). Standard RDA soybean management practices were applied for the cultivation. Days to 50% flowering (DF, about 50% of flowers open in all plants) and days to maturity (DM, approximately all pods are ripe; beans final color, dry and hard) of each KBSL were observed during cultivation, and 100-seed weight (SW) was measured after harvest. The harvested seeds were refrigerated at −20 °C until they were retrieved for analyses.

### 2.2. Phytochemical Analysis

In order to analyze the anthocyanin content in each KBSL, the hand-peeled seed coats (100 mg) of 172 KBSLs were mixed with 15 mL of 1% HCl in 99% MeOH for 24 h at 4 °C in the dark. After centrifugation at 13000 rpm for 10 min, each specimen was filtered through a 0.45 µm syringe filter and analyzed with an Agilent 1260 Infinity HPLC system (Agilent Technology, Santa Clara, CA, USA). The analysis was performed using a Waters XSelect HSS Cyano XP system (2.5 μm, 2.1 × 75 mm, Waters, Milford, MA, USA). The HPLC conditions were as follows: solvent A, 0.1% TFA/H2O; solvent B, 0.1% TFA/CH3CN; gradient, 5% (B) for 0.3 min, 20% (B) for 6.0 min, 95% (B) for 8.0 min, and 5% (B) for 10 min; column temperature, 40 °C; and flow rate, 0.5 mL/min. The filter detector was set at 520 nm (Supplemental Figure S1A).

To analyze the isoflavone content in each KBSL, 100 mg of each sample (whole seeds) was added to 2 mL of 80% MeOH and incubated with sonication for 1 h. The sample in each tube was hydrolyzed using 150 µL of 2N NaOH. After mixing for 10 min, the solutions were neutralized with 50 µl of glacial acetic acid. The sample was centrifuged for 5 min at 3000 rpm, and the collected supernatant was then filtered using a 0.45 µm syringe filter prior to analysis with an Agilent 1260 Infinity HPLC system (Agilent Technology). The analysis was performed using a Proshell 120 SB-C15 (2.7 μm, 2.1 × 50 mm, Agilent Technology). The HPLC conditions were as follows: solvent A, 0.1% TFA/H2O; solvent B, 0.1%TFA/CH3CN; gradient, 10% (B) for 0.35 min, 10%–30% (B) in 3.96 min, held at 30% (B) for 0.36 min, and re-equilibrated at 10% (B) for 1.8 min; column temperature, 30 °C; and flow rate, 0.58 mL/min. The filter detector was set at 254 nm (Supplemental Figure S1B).

### 2.3. Analysis of Antioxidant Activities

To compare antioxidant activities in 172 KBSLs, 2,2-diphenyl-1-picrylhydrazyl (DPPH), 2,2′-azino-bis-(3-ethylbenzothiazoline-6-sulfonic acid) (ABTS), ferric-reducing antioxidant power (FRAP), and total polyphenol content (TPC) assays were performed using previous methods described by Lee et al. [22]. To analyze the antioxidant activities, 100 mg of each KBSL powder (whole seed) was used. The absorbance at each assay was determined using a spectrophotometer (Epoch; Bio-Tek, Winooski, VT, USA).

### 2.4. Data Analysis

All the data collected from three replicate experiments were expressed as the mean ± standard deviation. Duncan’s multiple-range test and correlation analyses were used to determine the differences between the 172 KBSLs using SPSS Statistics 20 (SPSS Inc., Chicago, IL, USA). The DPPH results expressed as IC_50_ were converted to 1/IC_50_ before clustering analysis. Integration of the antioxidant capacity results derived from different chemical methods was used to calculate the relative antioxidant capacity index (RACI) [23]. Hierarchical clustering was performed using R statistical software [24]. PAST3 software was used for principal component analyses (PCA) [25]. 

## 3. Results

### 3.1. Agricultural Traits of 172 KBSLs

There were variations in the 172 KBSLs in days to 50% flowering (DF), days to maturity (DM), and 100-seed weight (SW) in 2012 and 2015 (Table 1 and Supplemental Table S1). The DF ranged from 49 to 79 days in 2012 and 56 to 70 days in 2015, with an average of 62.6 and 61.0 days, respectively. The DM ranged from 108 to 160 days in 2012 and 115 to 146 days in 2015, with an average of 139.5 and 138.2 days, respectively. The SW ranged from 10.3 to 40.0 g in 2012 and 9.7 to 54.8 g in 2015, with an average of 26.5 and 28.7 g, respectively. Among the three agricultural traits, the DF and SW were significantly different between black soybean accessions (*p* < 0.001) and experimental years (*p* < 0.001), whereas the DM was only significantly different between the accessions (*p* < 0.001) (Table 2).

### 3.2. Content of Phytochemicals in 172 KBSLs

The content of three anthocyanins (delphinidin-3-O-b-D-glucoside (D3G), cyanidin-3-O-b-D-glucoside (C3G), and petunidin-3-O-b-D-glucoside (Pt3G)) and isoflavone aglycones (daidzin, glycitin, and genestin) was measured in the 172 KBSLs (Table 1 and Supplemental Table S1). Among anthocyanins, the D3G, C3G, and Pt3G contents were 0.0 to 273.0, 12.2 to 2042.7, and 0.0 to 158.7 mg/100 g dried seed coat, respectively, in 2012 and 9.1 to 320.5, 51.9 to 1498.3, and 1.2 to 434.2 mg/100 g dried seeds, respectively, in 2015,. Among the anthocyanins, D3G and Pt3G showed significant differences between black soybean accessions (*p* < 0.001), the experimental years (*p* < 0.001), and the interaction between year and accessions (*p* < 0.001), whereas the C3G content was different between black soybean accessions (*p* < 0.001) and the interaction between year and accessions (*p* < 0.001) (Table 2).

In the three isoflavone aglycones, the contents of daidzin, glycitin, and genestin were 14.1 to 108.7, 0.8 to 43.2, and 15.6 to 115.6 mg/100 g dried seeds in 2012 and 4.6 to 81.0, 0.4 to 19.9, and 5.2 to 56.7 mg/100 g dried seeds in 2015, respectively. The three isoflavone contents were significantly different between the black soybean accessions (*p* < 0.001), experimental years (*p* < 0.001), and the interaction between year and accessions (*p* < 0.001). 

### 3.3. Antioxidant Activities of 172 KBSLs

**Figure 1:**
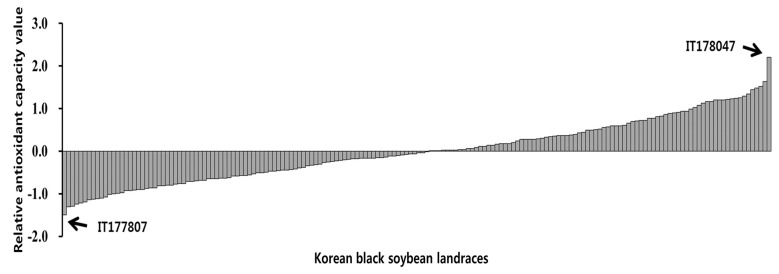
Relative antioxidant capacity index of 172 Korean black soybean landraces.

The DPPH radical-scavenging activity ranged from 63.7 to 311.1 (IC_50_) in 2012 and 16.4 to 154.6 (IC_50_) in 2015, with average values of 90.3 and 59.5 (IC_50_), respectively, among the accessions evaluated (Table 1 and Supplemental Table S1). The ABTS antioxidant activities of the 172 Korean black soybean accessions were 1.1 to 7.0 mg ascorbic acid equivalents (AAE)/g dried seeds in 2012 and 2.0 to 8.3 mg AAE/g dried seeds in 2015. The TPC ranged from 2.8 to 13.0 mg gallic acid equivalents (GAE)/g dried seeds in 2012 and 0.8 to 12.9 mg GAE/g dried seeds in 2015 with an average of 7.1 and 7.2 mg GAE/g, respectively. The FRAP assay revealed levels ranging from 0.3 to 2.5 mg AAE/g in 2012 and 0.3 to 3.1 mg AAE/g dried seeds, respectively. The DPPH, TPC, and FRAP were significantly different between the accessions (DPPH and FRAP, *p* < 0.001; TPC, *p* < 0.01), experimental years (*p* < 0.001), and the interactions between years and accessions (*p* < 0.01), respectively, whereas the ABTS activity was significantly different between black soybean accessions (*p* < 0.01) and the interaction between year and accessions (*p* < 0.01). In the results of RACI, IT178047 was the highest (2.21), followed by IT274457 (1.64), IT178132 (1.52), and IT177807 with the lowest value (−1.49) (Figure 1 and Supplemental Table S1).

### 3.4. Correlations between Six Phytochemicals in 172 Korean Black Soybean Landraces

The correlations between agricultural traits, phytochemicals, and antioxidant activities are shown in Table 3. There were positive correlations between anthocyanins (D3G and C3G, r = 0.461 (*p* < 0.001); D3G and Pt3G, r = 0.267 (*p* < 0.001); C3G and Pt3G, r = 0.287 (*p* < 00.01)) and isoflavones (daidzin and glycitin, r = 0.651 (*p* < 0.001); daidzin and genestin, r = 0.554 (*p* < 0.001); glycitin and genestin, r = 0.362 (*p* < 0.0001)). Between the anthocyanins and antioxidants, C3G showed correlations with DPPH (r = −0.194, *p* < 0.05), ABTS (r = 0.307, *p* < 0.001), TPC (r = 0.358, *p* < 0.001), and FRAP (r = 0.381, *p* < 0.001). D3G showed correlations with DPPH (r = −0.293, *p* < 0.001), ABTS (r = 0.199, *p* < 0.01), and FRAP (r = 0.199, *p* < 0.01), whereas Pt3G was not corrected with antioxidant activities. Among the isoflavones, only genestin showed a positive correlation with ABTS activities (r = 0.154, *p* < 0.05). Among the agricultural traits, the DF and DM showed correlations with anthocyanins, isoflavones, and antioxidant activity, whereas the SW was only correlated with isoflavones. 

### 3.5. PCA Analysis

PCA using the agricultural traits, phytochemicals, and antioxidant activities of 172 KBSLs indicated that five principal components (PCs) with eigenvalues >1 could explain 73.94% of the total variance (Table 4). The first PC, with an eigenvalue of 1.8138, explained 25.3% of the total variance. C3G was the variable with the largest positive loading. The second PC, with an eigenvalue of 1.526, explained an additional 17.9% of the total variance. Isoflavone was the variable with the largest negative loading. The third PC, with an eigenvalue of 1.235, explained an additional 11.7% of the total variance. Glycitin had the highest positive variance, and the SW had the highest negative variance. The fourth PC, with an eigenvalue of 1.187, explained an additional 10.8%. D3G had the highest positive variance. The fifth PC, with an eigenvalue of 1.028, explained an additional 8.0%. 

**Figure 2:**
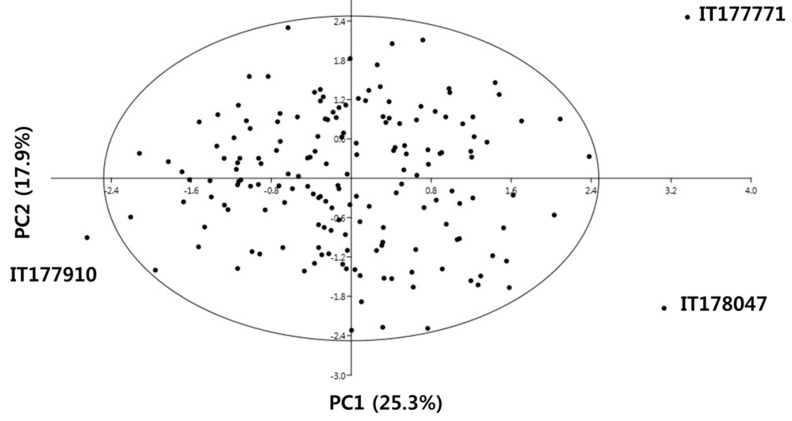
2D scatter diagram of principal component analysis (PCA) of 172 KBSLs based on agricultural traits, phytochemicals, and antioxidant activities.

The first two PCs are plotted in Figure 2. By placing an ellipse around the data representing the 95% confidence interval using Hoteling’s T2 statistic, it was possible to observe all KBSL, except for three landraces. Among the three landraces, IT177771 and IT178047 showed the highest Pt3G and daidzin content and D3G, C3G, ABTS, TPC, and FRAP, respectively, whereas IT177910 showed lower anthocyanin (D3G was not detected), isoflavone, and antioxidant activities.

### 3.6. Clustering Analysis

**Figure 3:**
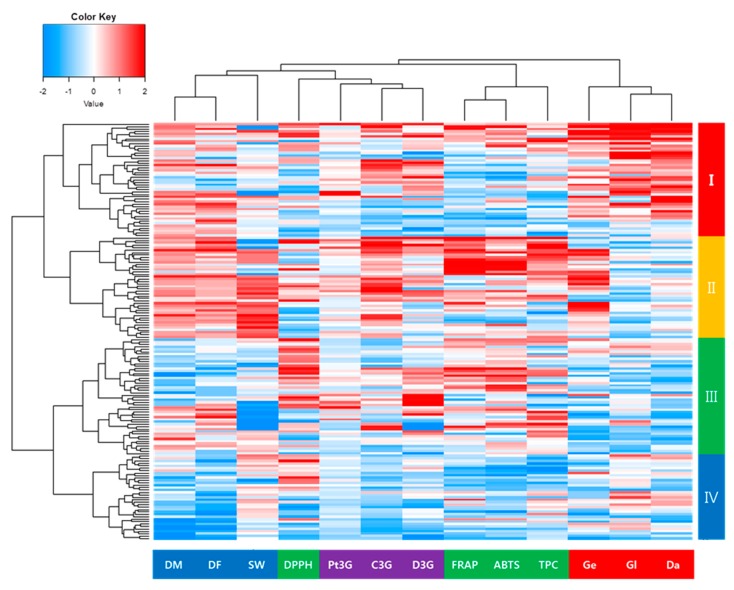
Hierarchical clustering analysis of agricultural traits, phytochemicals, and antioxidant activities in 172 Korean black soybean landraces.

The 172 KBSL were classified into four clusters according to their agricultural traits, phytochemicals, and antioxidant activities (Table 5 and Figure 3). Cluster I contained 47 KBSLs and had high isoflavone contents and the long DMs compared to the other clusters. Cluster II had 42 KBSLs and showed high anthocyanin contents, high antioxidant activities, long DFs and DMs, and high SWs. Cluster III consisted of 48 KBSLs and showed the lowest isoflavone content, high antioxidant activities, and lowest SWs. Cluster IV had 35 KBSLs with the lowest anthocyanin content and antioxidant activities.

## 4. Discussion

In this study, the agricultural traits, phytochemicals, and antioxidant activity of 172 KBSLs were evaluated to identify their potential as new breeding materials. The results showed that the 172 KBSLs had wide variations in agricultural traits, phytochemicals, and antioxidant activity. Hoisington et al. [26] suggested that the success of a breeding program depends on the genetic variability available to the breeders. In addition, Sthapit et al. [27] suggested that landraces could be effectively improved by simple trait selection if the landraces could offer sufficient natural variation in the population. The landrace contained important genetic variability, which determines their ability to adapt to changes in their environment and provides an important source of useful variability for breeding activities, provided that they are accompanied by information on characterization and agronomic evaluation [28,29]. Thus, the results of this study could be used as the basis for enhancing the availability of KBSLs. 

Many methods measuring antioxidant activity have been developed and used to determine the antioxidant capacity of plant extracts because they use relatively standard equipment and can deliver fast and reproducible results [30]. In this study, the free-radical-scavenging capacity of 172 KBSLs using the DPPH, ABTS, TPC, and FRAP assays was determined. Each method showed different grades depending on the soybean accessions and the year. Sun and Tanumihardjo [23] reported that each method of measuring antioxidant activity had its own limitations because various reaction mechanisms and different phase locations can affect the measurements. Therefore, Sun and Tanumihardjo [23] suggested using RACI. They described the advantage of RACI, which is a numerical scale that can integrate multiple chemical methods, thus allowing the comparison of the antioxidant capacity in a large number of samples. To compare data obtained by different chemical methods used to evaluate antioxidant activity, the RACI in the KBSLs was used in this study. The results of the RACI can be used to select KBSLs with high antioxidant activity so that new breeding materials can be confirmed.

The DF was positively correlated with anthocyanins, genestin, and antioxidant activities. Phommalath et al. [31] reported similar results, that flowering date showed significant positive correlations with TPC, total flavonoid content, and radical scavenging activity. They demonstrated that the reasons for the relationship between flowering dates and phenolic contents were due to the effect of temperature during maturation. Soybean accessions from high latitudes generally flower earlier than those from low latitudes because they are exposed to high temperatures during maturation. Tsukamoto et al. [32] reported that soybean seeds grown at high-temperature conditions had lower isoflavone contents than those grown at lower temperatures. Yaman et al. [33] and Cohen et al. [34] reported that higher temperatures inhibited the expression of flavonoid pathway genes, resulting in lower phenolic accumulation. The results of the present study may be attributed to similar mechanisms. In general, the monthly temperature in Korea is the highest in July and August. In fact, the average temperature in 2012 and 2015 was 24.4 and 26.4 °C in July, 25.2 and 25.4 °C in August, and 20.5 and 20.6 °C in September [35]. In this study, the DF in 2012 and 2015 was distributed between July 13 to August 12 and July 22 to August 5, and it is expected that the KBSL with an early DF might be exposed to higher temperatures during the maturation period. However, more research on the relationships between phenolic compounds and temperatures during maturation is needed.

The black soybeans exhibited high levels of antioxidant activity because of their high concentrations of phenolic compounds [36]. In addition, the phenolic compounds were affected by genotype and environment, as well as the interaction between genotype and environment [31,37]. In this study, there was a difference in antioxidant activities between the KBSLs and the years. It seemed that the difference in the antioxidant activities was due to differences in the polyphenolic contents. [38] reported that various antioxidants showed substantially varying antioxidant effectiveness in different systems due to different molecular structures. Due to difficulties in measuring each antioxidant component separately, and interactions between these different antioxidant components in the network, several methods have been developed to assess the total antioxidant capacity of all the nonenzymatic antioxidant components [39]. In this study, the DPPH, ABTS, and FRAP activities were positively associated with D3G and C3G, whereas isoflavone was not correlated with antioxidant activities (Table 3). The black pigmentation of black soybeans is due to the accumulation of anthocyanins in the epidermal palisade layer of the seed coat [40]. Anthocyanins in the seed coats play an important role in the protection against oxidative damage [41]. Among them, C3G, which was the most abundant anthocyanin found in black soybeans, exhibited strong antioxidant activity among the three anthocyanins isolated from black beans [42]. Lee et al. [43] reported that the black seed coats had significantly higher total phenolic and total anthocyanin contents and antioxidant activity compared to whole beans and dehulled beans

Genetic variability is important to the success of a breeding program [44]. Therefore, the collection and preservation of germplasm are important because it provides raw materials for plant breeding and crop improvement [45]. However, germplasm-only collections, without evaluation of their characteristics, are difficult to use by breeders who have different interests and possibly different requirements [46]. Whereas breeders focus on characteristics with perceived immediate value, other biologists may be more interested in potential variation or use the collections to better understand the properties and behavior of the plants [45]. In this study, 172 KBSLs were evaluated, which showed significant differences in agricultural traits, phytochemicals, and antioxidant activities. In particular, 42 KSBLs contained within cluster II had higher levels of phytochemicals and antioxidant activities compared to the other landraces. The results of this study could contribute to the more efficient conservation and utilization of KBSLs to broaden the genetic bases of commercially grown varieties of soybean.

## Figures and Tables

**Table 1:** Descriptive statistics of agricultural traits, phytochemicals, and antioxidant activity in 172 Korean black soybean landraces.

		Year	Min	Max	Mean	SD	Skewness	Kurtosis	CV(%)
Agricultural Traits	DF ^1^ (day)	2012	49	79	62.6	6.6	0.029	−0.409	10.6
		2015	56	70	61.0	3.2	0.687	−0.476	5.2
	DM (day)	2012	108	160	139.5	10.4	−0.559	0.153	7.4
		2015	115	146	138.2	8.5	−1.000	−0.169	6.1
	SW (g)	2012	10.3	40.0	26.5	8.1	−0.260	−0.580	30.5
		2015	9.7	54.8	28.7	8.5	−0.338	0.316	29.7
Anthocyanin (mg/100 g dried seed coat)	D3G	2012	0	273.0	92.7	65.5	0.636	−0.172	70.7
		2015	0	320.5	98.4	56.3	0.790	1.095	57.2
	C3G	2012	12.2	2042.7	509.4	378.8	1.140	1.259	74.4
		2015	51.9	1498.3	511.6	355.9	0.678	−0.549	69.6
	Pt3G	2012	0.0	158.7	17.8	17.4	3.850	25.386	97.7
		2015	0.0	434.2	18.5	34.4	10.502	126.198	185.8
Isoflavone (mg/100 g dried seeds)	Daidzin	2012	14.1	108.7	48.2	18.5	0.526	0.066	38.4
		2015	4.6	81.0	25.3	13.6	1.618	4.280	53.5
	Glycitin	2012	0.8	43.2	14.7	8.6	0.839	0.635	58.2
		2015	0.4	19.9	3.8	2.8	2.078	7.158	72.1
	Genestin	2012	15.6	115.6	54.1	20.0	0.650	0.134	36.9
		2015	5.2	56.7	30.0	10.9	0.017	−0.167	36.2
Antioxidant activity	DPPH (IC 50)	2012	63.2	311.1	108.0	45.3	1.514	2.705	42.0
		2015	16.4	154.6	59.5	29.7	0.940	0.588	49.8
	ABTS (mg AAE/g)	2012	1.1	7.0	5.0	1.2	−0.600	−0.183	23.7
		2015	2.0	8.3	4.9	1.6	0.392	−0.790	32.2
	TPC (mg GAE/g)	2012	2.8	13.0	7.1	2.3	0.425	−0.426	32.0
		2015	0.8	12.9	7.2	2.6	−0.176	−0.543	36.4
	FRAP (mg AAE/g)	2012	0.3	2.5	1.1	0.5	0.628	0.022	42.7
		2015	0.3	3.1	1.1	0.6	1.106	0.589	56.2

^1^ DF, days to 50% flowering; DM, days to maturity; SW, 100-seed weight; D3G, delphinidin-3-O-b-D-glucoside; C3G, cyanidin-3-O-b-D-glucoside; Pt3G, petunidin-3-O-b-D-glucoside; mg AAE/g, milligrams of ascorbic acid equivalents (AAE) per gram of dry weight sample; mg GAE/g, milligrams of gallic acid equivalents (GAE) per gram of dry weight sample.

**Table 2:** Mean squares according to year, genotypes and year x genotype interactions.

		Year (Y)	Genotype (G)	Interaction (G x Y)
Agricultural Traits	DF ^1^ (day)	13.1 ***	2.2 ***	-
	DM (day)	3.3 ns	2.9 ***	-
	SW (g)	52.5 ***	16.2 ***	-
Anthocyanin	D3G (mg/100 g)	60.8 ***	89.1 ***	60.7 ***
	C3G (mg/100 g)	0.6 ns	269.9 ***	85.49 ***
	Pt3G (mg/100 g)	23.8 ***	321.6 ***	235.1 ***
Isoflavone	Daidzin (mg/100 g)	26906 ***	198 ***	117.2 ***
	Glycitin (mg/100 g)	47810 ***	220.3 ***	159.1 ***
	Genestin (mg/100 g)	27837 ***	135.4 ***	152.7 ***
Antioxidant activity	DPPH (IC 50)	21647 ***	149.3 ***	151 ***
	ABTS (mg AAE/g)	1.6 ns	10.6 ***	3.9 ***
	TPC (mg GAE/g)	7.93 **	112.4 ***	18.8 ***
	FRAP (mg AAE/g)	60.32 ***	322.2 ***	218.4 ***

^1^ DF, days to 50% flowering; DM, days to maturity; SW, 100-seed weight; D3G, delphinidin-3-O-b-D-glucoside; C3G, cyanidin-3-O-b-D-glucoside; Pt3G, petunidin-3-O-b-D-glucoside; mg AAE/g, milligrams of ascorbic acid equivalents (AAE) per gram of dry weight sample; mg GAE/g, milligrams of gallic acid equivalents (GAE) per gram of dry weight sample; ns, not significant; **, *p* < 0.01; ***, *p* < 0.001.

**Table 3:** Correlations between agricultural traits, anthocyanins, isoflavones, and antioxidant activities in 172 Korea black soybean landraces.

	D3G ^1^	C3G	Pt3G	Daidzin	Glycitin	Genestin	DPPH	ABTS	TPC	FRAP	DF	DM
C3G	0.461 ***											
Pt3G	0.414 ***	0.287 ***										
Daidzin	0.045	0.049	0.179 *									
Glycitin	0.025	−0.069	0.159 *	0.651 ***								
Genestin	0.007	0.214 **	0.172 *	0.554 ***	0.362 ***							
DPPH	−0.293 ***	−0.194 *	−0.111	0.019	0.004	−0.041						
ABTS	0.199 **	0.307 ***	0.083	−0.047	−0.064	0.154 *	−0.247 ***					
TPC	0.133	0.358 ***	0.02	−0.074	−0.065	−0.011	0.054	0.471 ***				
FRAP	0.199 **	0.381 ***	0.055	−0.088	−0.024	0.141	−0.182 *	0.777 ***	0.533 ***			
DF	0.185 *	0.436 ***	0.253 ***	0.104	0.015	0.288 ***	−0.062	0.198 ***	0.173 *	0.195 *		
DM	0.169 *	0.445 ***	0.241 ***	0.296 ***	0.085	0.332 ***	0.023	0.153 *	0.123	0.181 *	0.704 ***	
SW	0	0.145	−0.062	0.175 *	−0.195 *	0.283 ***	0.109	−0.12	−0.083	−0.105	−0.015	0.264 ***

^1^ DF, days to 50% flowering; DM, days to maturity; SW, 100-seed weight; D3G, delphinidin-3-O-b-D-glucoside; C3G, cyanidin-3-O-b-D-glucoside; Pt3G, petunidin-3-O-b-D-glucoside; mg AAE/g, milligrams of ascorbic acid equivalents (AAE) per gram of dry weight sample; mg GAE/g, milligrams of gallic acid equivalents (GAE) per gram of dry weight sample; ^*, **, ***^ Pearson’s correlation *p*-value of < 0.05, 0.01, and 0.001, respectively.

**Table 4:** Principal component analysis of the phytochemicals and antioxidant activities of 172 Korea black soybean landraces, eigenvalues, and percentage variability explained by the first five components.

Parameter	PC1	PC2	PC3	PC4	PC5
Eigenvalue	1.813	1.526	1.235	1.187	1.028
% variance	25.3%	17.9%	11.7%	10.8%	8.1%
Cumulative variability	25.3%	43.2%	54.9%	65.8%	73.9%
D3G ^1^	0.281	0.069	0.110	0.507	−0.116
C3G	0.411	0.063	−0.186	0.167	−0.068
Pt3G	0.248	−0.127	0.145	0.453	0.167
Daidzin	0.151	−0.507	0.248	−0.167	−0.094
Glycitin	0.080	−0.393	0.502	−0.151	0.190
Genestin	0.253	−0.382	0.063	−0.236	−0.293
DPPH	0.134	0.150	0.353	0.342	−0.417
ABTS	0.346	0.310	0.189	−0.272	−0.157
TPC	0.270	0.286	−0.013	−0.336	0.170
FRAP	0.354	0.329	0.151	−0.304	−0.096
DF	0.359	−0.107	−0.278	0.066	0.402
DM	0.361	−0.229	−0.354	−0.004	0.199
SW	0.041	−0.212	−0.479	−0.064	−0.621

^1^ DF, days to 50% flowering; DM, days to maturity; SW, 100-seed weight; D3G, delphinidin-3-O-b-D-glucoside; C3G, cyanidin-3-O-b-D-glucoside; Pt3G, petunidin-3-O-b-D-glucoside; mg AAE/g, milligrams of ascorbic acid equivalents (AAE) per gram of dry weight sample; mg GAE/g, milligrams of gallic acid equivalents (GAE) per gram of dry weight sample.

**Table 5:** Average cluster values of agricultural traits, phytochemicals, and antioxidant activities of 172 Korea black soybean landraces.

Group	No. acc.	D3G ^1^ (mg/100 g)	C3G (mg/100 g)	Pt3G (mg/100 g)	Daidzin (mg/100 g)	Glycitin (mg/100 g)	Genestin (mg/100 g)
1	47	97.7a ^2^	514b	22.7a	51.0a	13.7a	47.4a
2	42	107.6a	816.2a	21.6a	33.6b	6.7c	48.2a
3	48	104.6a	440.7b	17.2ab	28.2c	7.1c	35.9b
4	35	65.7b	234.6c	9.2b	33.1b	9.3b	36.1b
	DPPH (IC_50_)	ABTS (mg AAE/g)	TPC (mg GAE/g)	FRAP (mg AAE/g)	DF (day)	DM (day)	SW (g)
1	80.2b	4.7b	6.9b	0.9c	63.4b	142.8a	26.8b
2	77.9b	5.5a	8.0a	1.4a	65.0a	145.1a	33.5a
3	67.6a	5.4a	7.4ab	1.2b	61.2c	135.9b	22.9c
4	80.2b	4.1c	6.1c	0.8c	56.6d	130.1c	28.0b

^1^ DF, days to 50% flowering; DM, days to maturity; SW, 100-seed weight; D3G, delphinidin-3-O-b-D-glucoside; C3G, cyanidin-3-O-b-D-glucoside; Pt3G, petunidin-3-O-b-D-glucoside; mg AAE/g, milligrams of ascorbic acid equivalents (AAE) per gram of dry weight sample; mg GAE/g, milligrams of gallic acid equivalents (GAE) per gram of dry weight sample. ^2^ The same letter in each column indicates no significant difference by Duncan’s multiple range test, *p* < 0.05.

## Supplementary Materials

The following are available online at https://www.mdpi.com/2076-3921/9/3/213/s1, Table S1. Agricultural traits, phytochemicals, and antioxidant activities of 172 Korean black soybean landraces. Supplemental Figure S1. HPLC chromatogram of anthocyanins (A) and isoflavones (B). D3G, delphinidin-3-O-b-D-glucoside; C3G, cyanidin-3-O-b-D-glucoside; Pt3G, petunidin-3-O-b-D-glucoside
