# Supplementary material for: Phytochemicals and Antioxidant Activity of Korean Black Soybean (Glycine max L.) Landraces
Source: Antioxidants (Basel). 2020 Mar 5;9(3):213. doi: 10.3390/antiox9030213 (PMC7139723; doi:10.3390/antiox9030213)
Supplement: Supplementary file 1 [file antioxidants-09-00213-s001.zip › Supplemental Figure.docx]

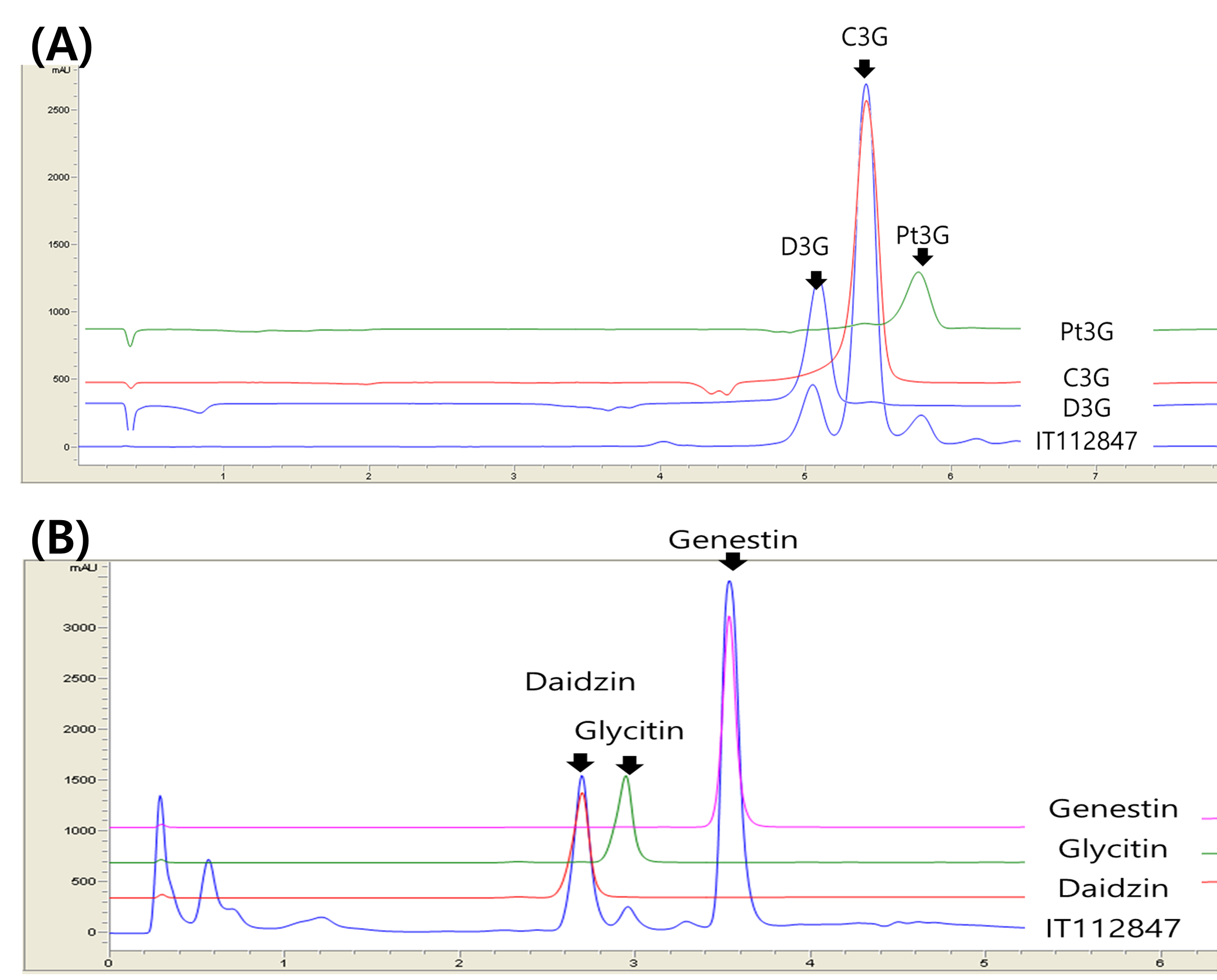


**Supplemental Figure 1.** HPLC chromatogram of anthocyanins (A) and isoflavones (B). D3G, delphinidin-3-O-b-D-glucoside; C3G, cyanidin-3-O-b-D-glucoside; Pt3G, petunidin-3-O-b-D-glucoside
